# In vitro gastrointestinal digestion methods of carbohydrate‐rich foods

**DOI:** 10.1002/fsn3.3841

**Published:** 2023-11-21

**Authors:** Rebeca Dávila León, Marcela González‐Vázquez, Karen Estefania Lima‐Villegas, Rosalva Mora‐Escobedo, Georgina Calderón‐Domínguez

**Affiliations:** ^1^ Escuela Nacional de Ciencias Biológicas Instituto Politécnico Nacional Ciudad de México Mexico

**Keywords:** carbohydrates, Infogest, in vitro digestion methods, RSIE, static methods

## Abstract

The trend toward healthier food products has led to an increase in the research of in vitro gastrointestinal digestion methods. Among the most used models, static models are the simplest. Most static models have three stages: oral, gastric, and intestinal, simulating the enzymatic, electrolyte, pH, temperature, and bile salt conditions. The studies that have taken the most notice are those related to antioxidant activity, followed by those dealing with proteins and carbohydrates using most of them static in vitro digestion models. The number of these studies has increased over the years, passing from 45 to 415 in a 10‐year period (2012–2023) and showing an interest in knowing the impact of food on human health. Nevertheless, published papers report different methodologies and analytical approaches. This review discusses the similarities and differences between the published static in vitro gastrointestinal digestion methods, with a focus on carbohydrates, finding that the most used protocol is Infogest, but with differences, mainly in the type of enzymes and their activity. Regarding in vitro gastrointestinal digestion of carbohydrates, many of the published studies are related to food and biomacromolecules, being the oral phase the most omitted, while the intestinal phase in the most diverse. Other methodologies to study the intestinal phase have been recommended, but the number of in vitro digestion studies using these methodologies (RSIE and BBMV) is still scarce but could represent a good alternative to analyze carbohydrates foods when combining with Infogest. More studies are required in this area.

## Graphical abstract

This review discusses the similarities and differences between the published static in vitro gastrointestinal digestion methods, with a focus on carbohydrates. The main differences are related to the type of enzymes and their activity, Infogest being the most complete methodology, but it does not consider the enzymes to hydrolyze sucrose, lactose, and trehalose, among others. Combining RSIE enzymes and Infogest method could represent a very good alternative to analyze carbohydrate foods.

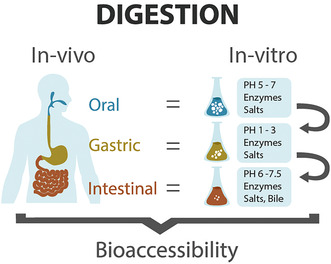

## 1 INTRODUCTION

Healthy food consumption has surged in many countries, as a result of the more interest of different age population on their own health, observed in the increase in global sales of fortified and functional food (Sloan, 2022) parallel to the increase of the gastrointestinal in vitro digestion studies that analyzed the nutraceutical capability of food and its behavior during gastrointestinal digestion. These methods have been employed in studying the mineral availability, beneficial and pathogenic microorganisms' survival rate, and other applications (Lucas González, 2016; Minekus et al., 2014; Xavier & Mariutti, 2021).

There are several ways of studying the in vitro gastrointestinal digestion that have been classified into static, dynamic, and semi‐dynamic methods (Brodkorb et al., 2019). The preference for these techniques over in‐vivo studies is related to the standardization of the methodology, the lack of inter‐ and intra‐individual variation, besides that they do not raise ethical concerns, have lower costs, and are less time‐consuming (Alegría et al., 2015; Hur et al., 2011; Lucas González, 2016; Nguyen et al., 2015), although they have trouble at providing equal results to in‐vivo processes, due to the complexity of the digestive process (Hur et al., 2011). However, the tendency to decrease this gap is increasing, as nowadays more recent approaches have been validated against in‐vivo data, providing equal results.

The static model is the simplest of the in vitro methods and comprised, most of them, three stages: oral, gastric, and intestinal, simulating enzymatic, electrolyte, pH, temperature, and bile salt conditions. These methods are usually carried out in flasks, so they are easy to use in most laboratories (Alegría et al., 2015; Xavier & Mariutti, 2021).

In contrast, dynamic models take into account for their simulation the flow of food entering each section of the gastrointestinal (GI) tract, pH gradients, the gradual addition of enzymes and gastric liquid, continuous gastric emptying, and peristalsis, among other processes during digestion. These models closely reproduce the conditions inside the human body, simultaneously resembling the mechanical and enzymatic transformations (Brodkorb et al., 2019; Thuenemann, 2015). Nevertheless, there are few studies with this type of model done to date (Guo et al., 2014; Guo et al., 2022; Egger et al., 2019; Li, Hu, et al., 2021). Some of them have been done using the *Human Gastric Simulator*, DIDGI®, or the *Bionic Gastrointestinal Reactor*, as well as some home‐made reactors (Brodkorb et al., 2019; Xavier & Mariutti, 2021). These dynamic models are relatively complex and expensive, in both installation and maintenance, as they require a large volume of reagents and equipment (Xavier & Mariutti, 2021).

There is a model in‐between static and dynamic, known as semi‐dynamic; it is based on the standardized static model but includes crucial kinetic aspects associated with the gastric phase, like gradual changes in pH, secretion, and constant fluid and enzyme drainage, making it more physiologically relevant than a static model (Hu et al., 2013; Mulet‐Cabero et al., 2020).

**FIGURE 1:**
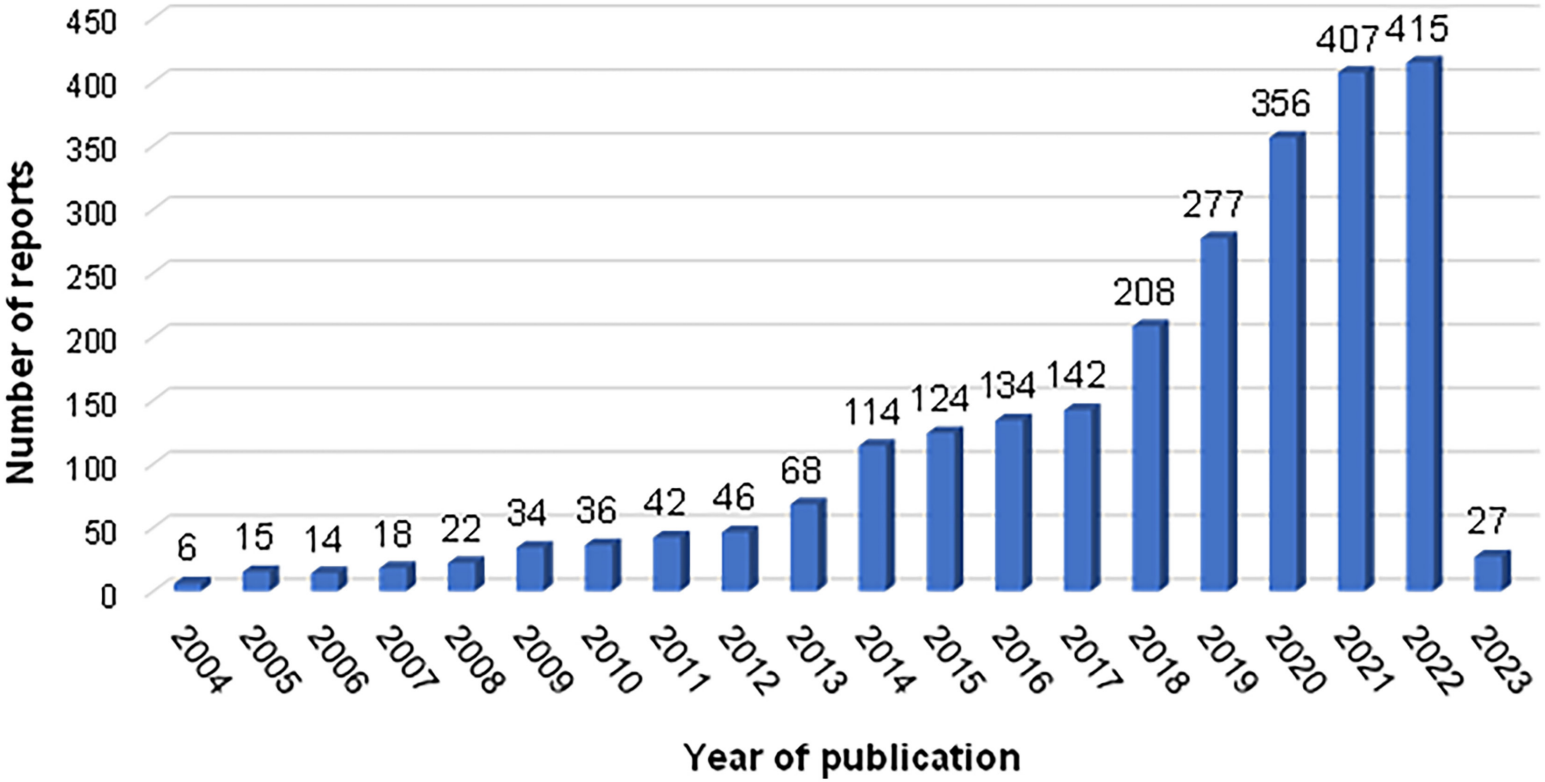
Articles related to in vitro gastrointestinal digestion (*Scopus* database), published in the last 20 years.

In the last years, the studies that include in vitro gastrointestinal digestion methods have been increasing (Figure 1), going from 46 reported articles in 2012 to 446 in 2022, this according to the Scopus database when searching for “in vitro digestion” and by narrowing the search with filters (keywords: “in vitro digestion” and “in‐vitro digestions”; year‐individual: 2004–2023) and limiting it to *articles* only.

**FIGURE 2:**
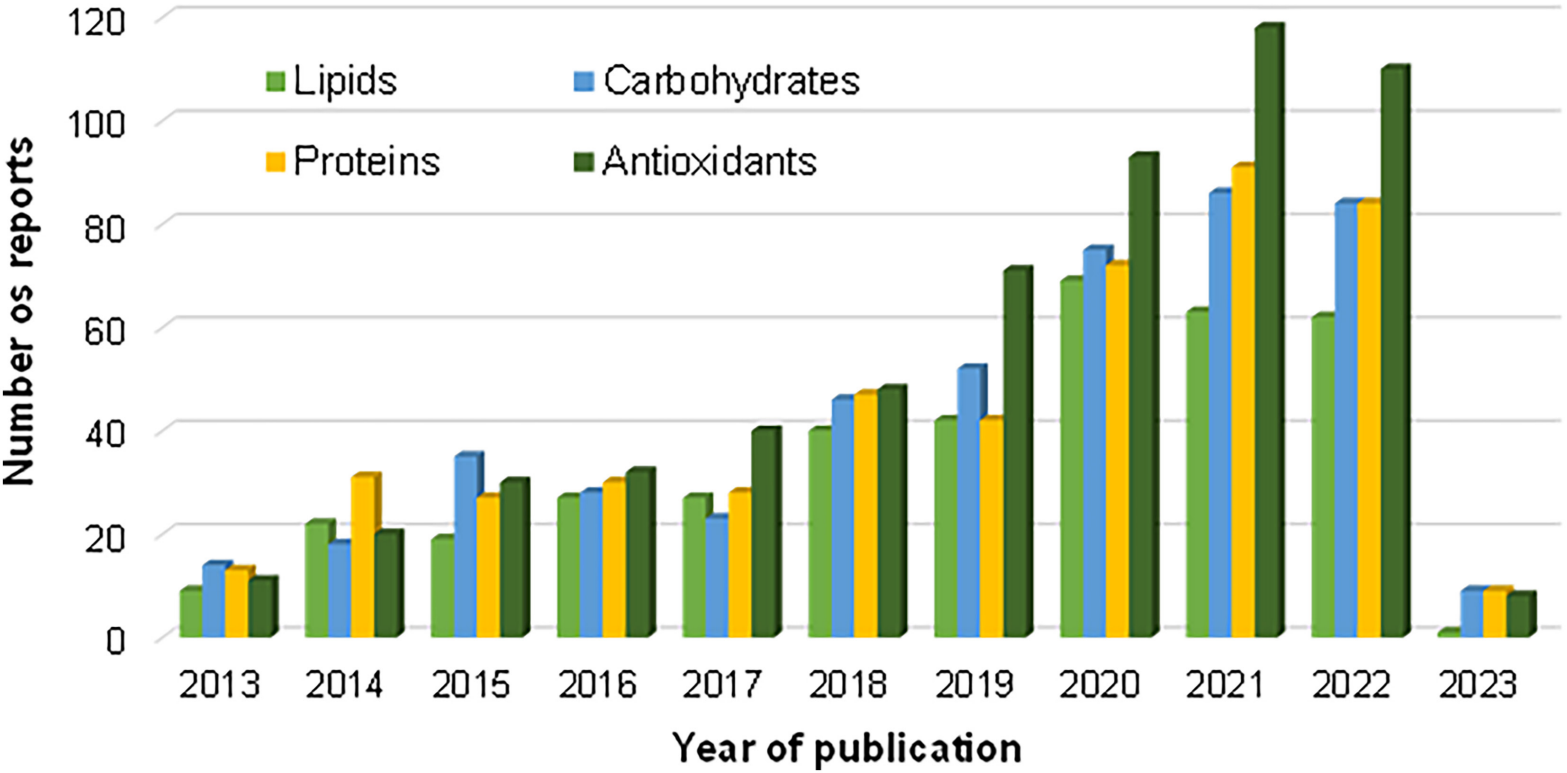
The trend in the number of publications related to in vitro gastrointestinal digestion in different food components (*Scopus* database).

Antioxidant activity has been the predominant topic in these reports, from 2013 to date (Figure 2). It was the most studied subject in 2016, followed by protein and carbohydrate‐related articles, which show almost the same increment.

It is also noted that in the last years (Figure 2) protein‐rich food studies increased, and the number of published articles doubled from 2019 to 2021, with carbohydrate in vitro digestion publications following the same trend, increasing from over 52 articles in 2019 to 86 in 2021. These numbers show the interest in knowing how food impacts human health. Nevertheless, published studies with the same components report differences in methodologies and analytical approaches; thus, this review aims to highlight the similarities and differences between in vitro digestion methods reported in the last 10 years, looking to lay the groundwork that will allow establishing a homogeneous technique with an application toward carbohydrates.

## 2 STATIC IN VITRO DIGESTION METHODS

**FIGURE 3:**
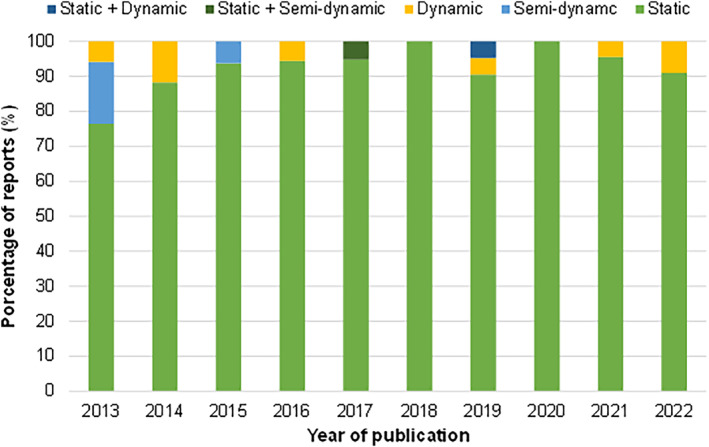
In vitro gastrointestinal digestion methods trend (2013–2022) (*Scopus* database, *n* = 11 to 23 per year).

As previously mentioned, static in vitro digestion models are the most reported (Brodkorb et al., 2019; Xavier & Mariutti, 2021); this trend has not changed in the last 10 years (Figure 3), as they are simpler and lower cost than dynamic methods, easy to evaluate and with high repeatability when standardized (Brodkorb et al., 2019).

## 3 DIGESTION PROTOCOLS

In static in vitro gastrointestinal digestion models, there is a huge protocol diversity with significant variables in the working parameters, such as pH values, length of each phase, types of enzymes used, agitation speed, and reagent quantities, which makes it difficult to compare the studies. Furthermore, some of these studies are based on fasting conditions, which is far from what occurs during food intake (Brodkorb et al., 2019; Li et al., 2020; Minekus et al., 2014). Nevertheless, their advantages (low cost, repeatability, etc.) make them widely accepted (Brodkorb et al., 2019; Minekus et al., 2014), above all when the matrix composition does not differ too much and the model is used only to analyze the endpoint of the digestion (Brodkorb et al., 2019). However, this level of variability led to the proposal of international harmonization, generating the Infogest protocol (Minekus et al., 2014) and its variants (Brodkorb et al., 2019; Xavier & Mariutti, 2021).

### 3.1 Infogest protocol

Infogest protocol was the outcome of an international network known as COST Action Infogest which, using in‐vivo digestion data, created an experimental protocol published in 2014 (Minekus et al., 2014). The main contributions of this protocol are the use of enzymes based on their activity and the incorporation of digestive fluids, developed using electrolytic solutions, enzymes, and CaCl_2_ (Xavier & Mariutti, 2021). The Infogest method was published by Minekus et al. in 2014 and was updated in 2019 by Brodkorb et al. as Infogest protocol 2.0. The main difference between Infogest and Infogest 2.0 is that in the latter all foods, be they liquids, semisolids, or solids, should pass through an oral phase, and that the use of gastric lipase in the stomach phase became mandatory (Xavier & Mariutti, 2021). Noteworthy, solid and semisolid foods can be shredded manually or electrically, in order to simulate chewing.

**FIGURE 4:**
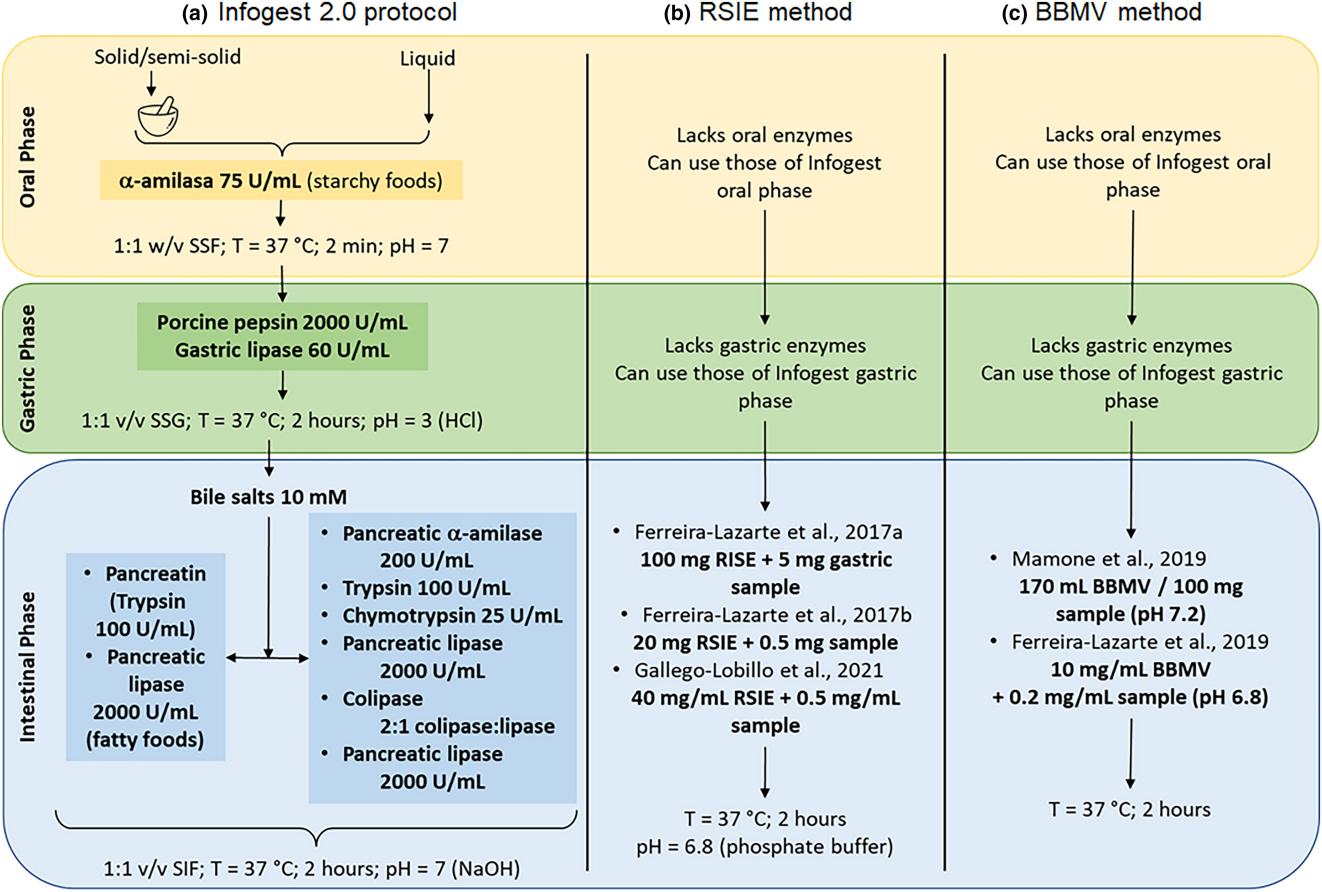
In vitro gastrointestinal digestion methods. (a) Infogest 2.0 protocol; (b) RSIE method; (c) BBMV method (Porcine brush border membrane vesicles). SGF, simulated gastric fluid; SIF, simulated intestinal fluid; SSF, simulated salivary fluid (Brodkorb et al., 2019; Minekus et al., 2014).

The Infogest method allows different alterations to accommodate study goals (Brodkorb et al., 2019) and shows good correlation in its endpoints, for each phase, with in‐vivo digestion data. However, at the intestinal level, this method focuses on starch hydrolysis by pancreatic α‐amylase (Brodkorb et al., 2019; Minekus et al., 2014) (Figure 4), without taking into account other enzymes that participate in the lysis of starch residues or other carbohydrates present in the food. The restricted addition of enzymes has been explained by the lack of standardization and agreement on enzyme activity and exposure time, and mentioned in the same protocol (Minekus et al., 2014). More recently, this shortcoming was considered in the RSIE method. To date, the Infogest method is one of the most trustworthy materials with starchy carbohydrates.

### 3.2 RSIE method

This method uses the small intestine of rats as an enzymatic source (RSIE rat small intestinal extract). It is used for the study of carbohydrate digestion based on the similarity between rat and human disaccharidase (glucoamylase, sucrase, trehalase, and lactase) activity. This method shows a high correlation with in‐vivo digestion data (Ferreira‐Lazarte, Olano, et al., 2017; Ferreira‐Lazarte et al., 2020) and has demonstrated the hydrolysis, even partial, of several carbohydrates that were thought to arrive intact to the colon, like fructans and fructooligosaccharides (Ferreira‐Lazarte, Montilla, et al., 2017; Ferreira‐Lazarte, Olano, et al., 2017; Ferreira‐Lazarte et al., 2020; Gallego‐Lobillo et al., 2021; Lee et al., 2016; Seo et al., 2020).

RSIE has been used in the study of α‐glucosidase inhibition, with the purpose of decreasing the hydrolysis and excessive absorption of carbohydrates in the small intestine (Ohta et al., 2002; Oki et al., 1999; Shinde et al., 2008; Zhang et al., 2011). Nevertheless, the current methodology shows that there are several drawbacks, mainly regarding enzymatic activity (Ferreira‐Lazarte, Montilla, et al., 2017; Ferreira‐Lazarte, Olano, et al., 2017; Gallego‐Lobillo et al., 2021; Kajszczak et al., 2022; Lee et al., 2016; Seo et al., 2020; Shin et al., 2019). The hydrolysis temperature (37°C) has been the only parameter in common between different reports. This lack of homogeneous working conditions is outstanding and should be considered, mainly the lack of standardization on activity from brush border enzymes, as well as that rats' small size and their anatomic and physiologic difference from humans can be a challenge for their use (Ferreira‐Lazarte et al., 2020), without forgetting that this rodent is granivore, does cecum fermentation and caecotrophy (Heinritz et al., 2013). Despite these drawbacks, it has been demonstrated that the RSIE method produces reliable results.

### 3.3 Pig small intestine enzymes method

Another proposal to evaluate foods rich in non‐starchy carbohydrates is to use pig small intestine enzymes, as these mammals have some similitude with humans, such as their anatomy, physiology, nutrient requirements, gut microbial systems, and similar intestinal problems, as well as being a food source that reduces ethical issues and is referred to in the field of health (Ferreira‐Lazarte et al., 2020; Heinritz et al., 2013). Pig's main microbiotas are phyla Firmicutes and Bacteroidetes (Heinritz et al., 2013).

The use of enzymes sourced from the small intestine of pigs is considered trustworthy, as it entails a robust model for in vitro digestion and colonic studies, and although it can also be compared to human assays, their use is not widespread. The most used of these enzymes is pig pepsin, not to be confused with pig small intestine extract. For the study of carbohydrates, glycoproteins (intestine disaccharidases) can be used; they are attached to the apical membrane, which can be detached as brush border membrane vesicles (BBMV) (Ferreira‐Lazarte et al., 2020; Hooton et al., 2015). Nevertheless, the number of published articles using this methodology for in vitro hydrolysis is minimal (Ferreira‐Lazarte et al., 2019; Mamone et al., 2019). There is quite a large gap between the experimental conditions reported by both authors, preventing a clear picture of ideal hydrolysis conditions from coming to light. In Figure 4, we compare the three static digestion methods.

It is important to mention that for RSIE and BBMV methods there is not a standardized protocol and that they are only used in the intestine step, so it is important to establish not only the weight of the intestinal extract but also the activity of each enzyme involved, as well as the length and speed of the process. For this purpose, Infogest protocol can be used as the foundation.

## 4 STATIC IN VITRO DIGESTION METHOD VARIANTS

**FIGURE 5:**
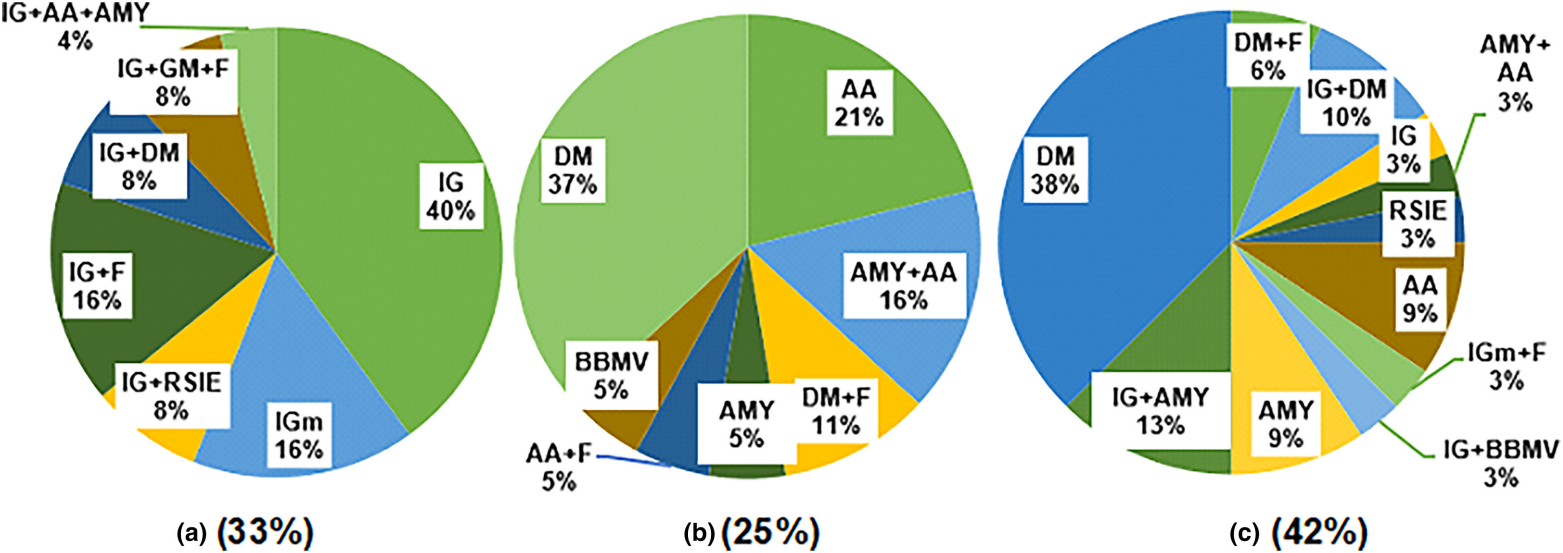
Static in vitro gastrointestinal digestion methodology variants. (a) Infogest method with or without modification. (b) Methods other than Infogest. (c) Methods without enough information to replicate in vitro digestion; AA, α‐amylase addition; AA+F, α‐amylase addition and colonic fermentation; AMY + AA, Amyloglucosidase addition and α‐amylase; AMY, Amyloglucosidase addition; DM + F, Different model to Infogest and colonic fermentation; DM, Different model to Infogest; IG + AA+AMY, Infogest pulse amylase and amyloglucosidase; IG + DM + F, Infogest with different methods and colonic fermentation; IG + DM, Infogest + different reported methods; IG + F, Infogest and colonic fermentation; IG + RSIE, Infogest and RSIE; IG, Infogest without modifications; IGm, Infogest with modifications; RSIE, rat small intestine extract.

Focusing on carbohydrates, among the static in vitro gastrointestinal digestion studies, from 2017 to 2023 first months (76 references), 33% of them used Infogest protocol with or without methodology variants (Egger et al., 2019; Igual et al., 2022; Iqbal et al., 2021; Lestido‐Cardama et al., 2022; Lin et al., 2021; Lucas‐González et al., 2021; Skalickova et al., 2022; Sousa et al., 2020; Varnaitė et al., 2022; Wei et al., 2020). The reported variants included a mix of RSIE with colonic fermentation (Aguirre‐Calvo et al., 2020; Bai et al., 2021; Chait et al., 2020; Liu, Li, et al., 2021), or mainly enzyme addition (Figure 5a).

The models that use a different methodology than Infogest (Figure 5b) are 25% of the total, where 9% used the same enzymes reported by Infogest, but with differing enzymatic activity (Blanquet‐Diot et al., 2021; Hayder et al., 2022; Joehnke et al., 2021; Korompokis et al., 2021; Schmid et al., 2022; Xu et al., 2021), while the rest added other enzyme types. Different published studies lack the necessary information in order to replicate the experiments (Figure 5c), like digestion fluid composition, temperature, pH values, step length, enzyme types, and activity, representing 42% of the sample.

## 5 IN VITRO DIGESTION OF CARBOHYDRATES

Polysaccharide digestion in the intestine is done by different enzymes that break them down into monosaccharides. The enzymes that are involved in this process are salivary or pancreatic α‐amylases, α‐glucosidases, and β‐glucosidases located in the brush border small intestine cells, where most carbohydrate absorption and hydrolysis occur (Ferreira‐Lazarte et al., 2020; Julio‐Gonzalez et al., 2019; Welcome, 2018).

Salivary and pancreatic α‐amylases break α‐1,4 starch bonds that are away from the chain ends and branching starts, releasing maltose, maltotriose, and α‐dextrins, carbohydrates that are then hydrolyzed by enzymes in the brush border small intestine cells (Butterworth et al., 2011; Prieto Bozano & Fernández Caamaño, 2014), mainly trehalase, lactase, and the protein complexes maltase‐glucoamylase (amyloglucosidase or MGAM) and sucrase‐isomaltase (SI) (Hooton et al., 2015; Welcome, 2018). Lactase is responsible for all intestinal activity on lactose, just as trehalase is responsible for trehalose digestion. Nevertheless, most in vitro gastrointestinal digestion studies do not include these enzymes. For example, Liu, Li et al. (2021) consider that all polysaccharides present in *Oudemansiella radicata* are not digestible by digestion enzymes, despite trehalose being usually present in said fungus (Ingold & Hudson, 1993). Egger et al. (2019), Ferreira‐Lazarte, Montilla, et al. (2017), and Mudgil et al. (2022) worked with milk, that contains lactose, but only Ferreira‐Lazarte, Montilla, et al. (2017) added an enzymatic solution that could have added lactase. In both cases (trehalose and lactose), the effect of these disaccharides and hydrolysis products is not considered in the Infogest method, underestimating these carbohydrates' bioaccessibility. However, techniques such as the use of Caco‐2 cells and Ussing chamber (Álvarez‐Olguín et al., 2022; Kalungwana et al., 2023; Larsen et al., 2001; Ozorio et al., 2020) have been proposed to overcome this disadvantage for trehalose and lactose bioabsorption evaluation.

This trend is present in several in vitro gastrointestinal digestion studies where the enzymatic activity of α‐glucosidases is not taken into account, as the MGAM. This enzyme hydrolyzes the 1,4 end residues of successively linked α‐D‐glucose molecules, starting from their non‐reducing ends, creating β‐D‐glucose as the final product. In addition, it breaks α‐1,6 bonds between glucose molecules. And on the other hand, the sucrase‐isomaltase, an α‐1,2, α‐1,4, and α‐1,6 glycosidic bond hydrolyzing enzyme, produces mainly glucose and fructose (Ferreira‐Lazarte et al., 2020; Lee et al., 2016; Prieto Bozano & Fernández Caamaño, 2014; Welcome, 2018).

From this, we can conclude that static in vitro gastrointestinal digestion focused on carbohydrate‐rich foods should be as similar as possible to in‐vivo gastrointestinal digestion, considering as many enzymes involved in the biological process as possible, as disregarding them might result in overestimating the proportion of non‐digestible carbohydrates.

## 6 IN VITRO GASTROINTESTINAL DIGESTION ENZYMES USED IN PRODUCTS WITH CARBOHYDRATES IN THEIR COMPOSITION

Over the last few years, many of the publications on in vitro gastrointestinal digestion of carbohydrates (Table 1) have focused on foods (53%), mainly cereals and legumes, followed by isolated biomacromolecules (32%) such as starch, proteins, and fiber. Encapsulated foods (11%) have been studied too, as well as non‐food products like bisphenol (Lestido‐Cardama et al., 2022), metallic nanoparticles, and methylcobalamin (Hayder et al., 2022) for food additives. In vitro oral digestion phase has been omitted in 55% of these studies and, when present, has involved the use of several types of alpha‐amylases, varying in their activity from 75 U/mL (Lin et al., 2021) to 1500 U/mL (Mamone et al., 2019), as well as in their origin, with enzymes coming from *Bacillus* sp. (Lestido‐Cardama et al., 2022), humane saliva (Makran et al., 2022), pig saliva (Garvey et al., 2022), pig pancreas (Singh et al., 2020), and *Aspergillus oryzae* (Somaratne et al., 2019). The lack of an oral phase could be considered to have little effect on the hydrolytic process because it is a short phase (2 min) with minimal alpha‐amylase activity, besides it being added again during the intestinal phase.

**TABLE 1:** Use percentages of enzymes and other components in different in vitro gastrointestinal digestion phases.

Reports (%)		Enzymes (%)																			
		Oral		Gastric				Intestine													
		α‐Amylase		Pepsin		Lipase		P		B		PE		Amg		RSIE		BBMV		Others	
		With	Without	With	Without	With	Without	With	Without	With	Without	With	Without	With	Without	With	Without	With	Without	With	Without
Food^a^	53	44	56	82	18	15	85	59	41	36	64	41	59	18	82	3	97	0	100	5	95
Biocomponents^b^	32	52	48	74	26	13	87	74	26	52	48	17	83	30	70	4	96	9	91	0	100
Encapsulated^c^	11	25	75	88	13	0	100	63	38	88	13	25	75	0	100	0	100	0	100	0	100
Not‐food‐C^d^	4	67	33	100	0	0	100	100	0	33	67	0	100	0	100	0	100	0	100	0	100
Global	100	45	55	81	19	12	88	66	34	47	53	30	70	19	81	3	97	3	97	3	97

Abbreviations: Amg, amyloglucosidase, alpha‐glucosidase; B, Bile salts; BBMV, pig brush border membrane vesicles; Not‐food‐C, Not food components; Others, invertase, cholesterol esterase; P, pancreatin (trypsin, lipase, amylase); PE, independently added Pancreatic enzymes (trypsin, chymotrypsin, lipase, amylase); RSIE, rat small intestine extract (trehalase, MGAM, SI and lactase).

^a^ Alongi et al., 2019; Asensio‐Grau et al., 2021; Atzler et al., 2021; Cervini et al., 2021; Chang et al., 2021; Di Cairano et al., 2022; Edwards et al., 2021; Fekri et al., 2020; Graça et al., 2021; Huang et al., 2021; Lachowicz et al., 2021; Li, Li et al., 2021; Lyu et al., 2022; Ma et al., 2019; Nadia et al., 2022; Peng et al., 2022; Perales‐Vázquez et al., 2020; Rocchetti et al., 2020; Swackhamer et al., 2022; Szwajgier et al., 2019.

^b^ Di et al., 2018; Gong et al., 2019; Li, Gilbert et al., 2021; Liu, Du et al., 2021; Lyu et al., 2021; Mahajan et al., 2022; Peressini et al., 2020; Szwajgier et al., 2021; Tornero‐Martínez et al., 2019; Vamanu et al., 2020; Wu, Fu et al., 2021; Wu, Yuang et al., 2021.

^c^ Ahmad & Gani, 2021; Vonghirundecha et al., 2022; Xue et al., 2022.

^d^ Un et al., 2022.

In the gastric digestion phase (Table 1), most studies quote the use of pepsin (81%), mainly from pigs (Ding et al., 2021; Hayder et al., 2022; Lee et al., 2020), with a common concentration of 2000 U/mL (Egger et al., 2019; Sousa et al., 2020; Xu et al., 2021) and in a few cases (12%) lipases are added (Garvey et al., 2022; Lee et al., 2020;Makran et al., 2022; Skalickova et al., 2022) which, like amylases in the oral phase, are from a different origin, mainly fungal (Lee et al., 2020; Somaratne et al., 2019), yeasts (Garvey et al., 2022), and rabbit (Makran et al., 2022; Skalickova et al., 2022), and their activity ranges from 20 U/mL (Li, Hu et al., 2021) to 120 U/mL (Somaratne et al., 2019). The omission of these lipases can be related to the kind of product studied, as many carbohydrate studies following the in vitro gastrointestinal digestion are carried out in low‐fat samples, built mainly from carbohydrates. Nevertheless, it is recommended that the addition of this enzyme be included in the protocol, as it is part of in‐vivo gastrointestinal digestion and several carbohydrate‐based products have fats in their content.

The in vitro digestion intestinal phase is the one that uses the most enzymes, pancreatin being the most used, at an average concentration of 100 trypsin U/mL (Egger et al., 2019; Gallego‐Lobillo et al., 2021; Gallo et al., 2022; Lestido‐Cardama et al., 2022; Sousa et al., 2020; Varnaitė et al., 2022). In some articles (30%), instead of using a pancreatic solution of mainly trypsin, chymotrypsin, lipases, or α‐amylases, they are added independently but not as a whole. These studies describe enzyme concentrations in a partial way, so it is difficult to replicate the experiments. Amyloglucosidase (19%) has been reported in many foods and biocomponent studies, used in a solution range from 27 U/mL (Kan et al., 2020) to 3000 U/mL (Wang et al., 2021), with the concentration being often‐times not mentioned.

All of these enzymes (trypsin, lipase, and amylase) are part of the Infogest in vitro gastrointestinal digestion protocol (Minekus et al., 2014). However, during in vivo gastrointestinal digestion several other enzymes, such as MGAM, SI, trehalase, and lactase, that are not considered in such protocol are present. These are highly important for the study of carbohydrate‐based products. These enzymes are considered in RSIE and BBMV methods, but the articles that mention them are scarce (3%).

In the RSIE reports, Ferreira‐Lazarte, Olano, et al. (2017) evaluated the digestibility of lactulose, fructooligosaccharides (FOS), and galactooligosaccharides (GOS), where the addition of rat intestine enzymes generated similar results to in‐vivo gastrointestinal digestion. The same authors analyzed the effect of in vitro gastrointestinal digestion using the same method in milk added with GOS and lactulose and reported that GOS were considered not digestible by Infogest but presented hydrolysis under RSIE. Following a similar set of assays, Gallego‐Lobillo et al. (2021) report a similar behavior with pectin use. These results show that it is recommended to include rat intestine or pig brush border small intestine cells when carbohydrates are analyzed, to get results that more closely resemble in‐vivo gastrointestinal digestion.

## 7 CONCLUSION

The study of food in vitro gastrointestinal digestion methods has grown in the last decade, where a tenfold increment has been observed, being antioxidant activities, the predominant topic followed, almost, doubling the number of published studies from 2019 to 2021. Regarding the type of methodology, static in vitro digestion models are the most reported without changing the trend in the last 10 years. Nevertheless, published studies showed differences in methodologies and analytical approaches for similar food products. From 2017 to 2023 first months, one‐third of static gastrointestinal digestion studies used in this study reported Infogest protocol as the base methodology with or without variations, one quarter used a different method, while almost half of the published studies lack the necessary information in order to replicate the experiments. Regarding in vitro gastrointestinal digestion of carbohydrates, half of the studies of this review were focused on foods, followed by isolated biomacromolecules, being the oral phase the most omitted and in many cases, the activity and source of enzymes involved were different. The gastric digestion phase was the most homogeneous, in both the type and enzyme concentration, while the intestinal phase was the most diverse, still being the Infogest protocol the most reported and accurate method. Nevertheless, when studying carbohydrate‐based foods, the addition of enzymes, such as MGAM, SI, trehalase, and lactase, was recommended, but considering enzymatic activity as well as operating conditions would allow a better understanding of carbohydrate hydrolysis. More studies are required in this area.

## AUTHOR CONTRIBUTIONS

**Rebeca Dávila León:** Conceptualization (equal); data curation (equal); formal analysis (equal); investigation (equal); methodology (equal); writing – original draft (equal). **Marcela González‐Vázquez:** Data curation (equal); formal analysis (equal); investigation (equal); supervision (equal). **Karen Estefania Lima‐Villegas:** Data curation (equal); formal analysis (equal); investigation (equal). **Rosalva Mora‐Escobedo:** Formal analysis (equal); funding acquisition (equal); project administration (equal); resources (equal). **Georgina Calderón‐Domínguez:** Conceptualization (equal); funding acquisition (equal); investigation (equal); project administration (equal); resources (equal); software (equal); supervision (equal); visualization (equal); writing – review and editing (equal).

## FUNDING INFORMATION

This research was funded through projects: 20210624, 20221471, and 20230985 from the Instituto Politécnico Nacional (IPN, Mexico).

## CONFLICT OF INTEREST STATEMENT

The authors confirm that they have no conflict of interest to declare for this publication.

## Data Availability

The authors confirm the availability of the data and material.
